# Post Transplantation Bilirubin Nanoparticles Ameliorate Murine Graft Versus Host Disease *via* a Reduction of Systemic and Local Inflammation

**DOI:** 10.3389/fimmu.2022.893659

**Published:** 2022-06-01

**Authors:** Sumedha Pareek, Alexandra S. Flegle, Drew Boagni, Jin Yong Kim, Dohyun Yoo, Abel Trujillo-Ocampo, Sung-Eun Lee, Mao Zhang, Sangyong Jon, Jin S. Im

**Affiliations:** ^1^ The University of Texas MD Anderson Cancer Center, UTHealth Graduate School of Biomedical Sciences, Houston, TX, United States; ^2^ Department of Hematopoietic Biology and Malignancy, Division of Cancer Medicine, MD Anderson Cancer Center, Houston, TX, United States; ^3^ Graduate School of Medical Science and Engineering, Korea Advanced Institute of Science and Technology (KAIST), Daejeon, South Korea; ^4^ Institute for the BioCentury, Department of Biological Sciences, Korea Advanced Institute of Science and Technology (KAIST), Daejeon, South Korea; ^5^ Department of Hematology, Seoul St. Mary’s Hospital, College of Medicine, Seoul, South Korea; ^6^ Department of Stem Cell Transplantation and Cellular Therapy, Division of Cancer Medicine, MD Anderson Cancer Center, Houston, TX, United States

**Keywords:** bilirubin, nanomedicine, graft-versus-host-disease (GVHD), anti-inflammatory therapy, nanoparticles, leukemia

## Abstract

Allogeneic stem cell transplantation is a curative immunotherapy where patients receive myeloablative chemotherapy and/or radiotherapy, followed by donor stem cell transplantation. Graft versus host disease (GVHD) is a major complication caused by dysregulated donor immune system, thus a novel strategy to modulate donor immunity is needed to mitigate GVHD. Tissue damage by conditioning regimen is thought to initiate the inflammatory milieu that recruits various donor immune cells for cross-priming of donor T cells against alloantigen and eventually promote strong Th1 cytokine storm escalating further tissue damage. Bilirubin nanoparticles (BRNP) are water-soluble conjugated of bilirubin and polyethylene glycol (PEG) with potent anti-inflammatory properties through its ability to scavenge reactive oxygen species generated at the site of inflammation. Here, we evaluated whether BRNP treatment post-transplantation can reduce initial inflammation and subsequently prevent GVHD in a major histocompatibility (MHC) mismatched murine GVHD model. After myeloablative irradiation, BALB/c mice received bone marrow and splenocytes isolated from C57BL/6 mice, with or without BRNP (10 mg/kg) daily on days 0 through 4 post-transplantation, and clinical GVHD and survival was monitored for 90 days. First, BRNP treatment significantly improved clinical GVHD score compared to untreated mice (3.4 vs 0.3, p=0.0003), and this translated into better overall survival (HR 0.0638, p=0.0003). Further, BRNPs showed a preferential accumulation in GVHD target organs leading to a reduced systemic and local inflammation evidenced by lower pathologic GVHD severity as well as circulating inflammatory cytokines such as IFN-γ. Lastly, BRNP treatment post-transplantation facilitated the reconstitution of CD4^+^ iNK T cells and reduced expansion of proinflammatory CD8α^+^ iNK T cells and neutrophils especially in GVHD organs. Lastly, BRNP treatment decreased ICOS^+^ or CTLA-4^+^ T cells but not PD-1^+^ T cells suggesting a decreased level of T cell activation but maintaining T cell tolerance. In conclusion, we demonstrated that BRNP treatment post-transplantation ameliorates murine GVHD *via* diminishing the initial tissue damage and subsequent inflammatory responses from immune subsets.

## Introduction

Allogeneic stem cell transplantation (ASCT) is a curative immunotherapy where patients receive ablative chemotherapy and/or radiation followed by donor stem cell infusion, and works through varying degree of anti-tumor effects of donor T cells (1). Acute Graft Versus Host Disease (GVHD) due to dysregulated donor T cells, is the major complication that occurs in 50-80% of patients with ASCT and is often fatal without upfront aggressive treatment (1). Systemic corticosteroids remain as the first line of therapy however only 50-80% of patients respond. Although ruxolitinib (JAK2 inhibitor) has been recently approved as second line therapy, the outcome for patients with steroid refractory disease remains poor (1–4). Therefore, new and innovative approach to prevent and treat acute GVHD is much needed to reduce post-transplantation mortality and morbidity.

Acute GVHD post-transplantation is thought to start from the tissue injury mediated by conditioning regimen consisting of chemotherapy and/or radiation (5, 6). Once damaged tissue releases damage-associated molecular patterns (DAMPs), chemokines, and gut commensals, the innate immunity is activated to initiate the inflammatory milieu. Concurrently, host and recipient antigen presenting cells prime donor T cells *via* cross-presentation of allo-antigens released from damaged tissue and recruit various immune cells that eventually promote strong Th1/Th17 cytokine storm further escalating tissue damage in skin, gut, and liver, the major organs affected in acute GVHD (5, 6). The majority of pharmacological and cellular therapeutics currently under active investigation attempt to target events in the later stage of acute GVHD cascade such as dysregulated donor T cells and their metabolism, inflammatory cytokines, and immune cell trafficking, or to promote global immune-regulation *via* expansion of regulatory T cells (2, 5–9). However, none of these approaches focus on minimizing tissue damage, the very initial event opening the acute GVHD cascade.

Bilirubin nanoparticles (BRNP) are water-soluble PEGylated bilirubin that preferentially accumulate at the site of inflammation with leaky vasculature and exert potent anti-inflammatory property through the ability to scavenge reactive oxygen species generated during inflammation (10–16). They have been recently shown to effectively prevent the disease process in hepatic injury in ischemic-reperfusion animal model (10, 13), asthma (11), dextran sodium sulfate induced inflammatory bowel disease (IBD) murine model (12), pancreatic islet xenotransplantation (16), bleomycin induced pulmonary fibrosis (14), and experimental autoimmune encephalitis (15). As the pathophysiology of acute GVHD involves the inflammatory milieu occurred in damaged tissue by conditioning regimen, we investigated whether BRNP treatment can selectively accumulate at the site of inflammation and prevent acute GVHD in murine major histocompatibility (MHC) mismatched bone marrow transplantation model.

## Materials and Methods

### Materials

The following antibodies were purchased from Biolegend or BD Bioscience: B220-Pacific Blue (RA3-6B2), CD3e-FITC & APC-Cy7 (145-2C11), CD4-APC (GK.1.5), CD8α-BUV395 & PE (53-6.7), CD11b-PerCP Cy5.5 (M1/70), CD11c-BUV786 (N418), CD25-APC-Cy7 (3C7), CTLA-4-PE (UC10-4F10-11), Gr-1-PE-Cy7 (RB6-8C5), ICOS-BUV510 (C398.4a), Ly6C-BUV510 (HK1.4), Ly6G-APC (1A8), MHC II (1-A^b^)-FITC (KH74), MHC II (1-A^d^)-FITC (39-10-8), and PD1-BUV786 (29F.1A12). αGalCer/mCD1d tetramers were provided by NIH tetramer facility.

### Animal Studies

All animal experiments were conducted per The University of Texas MD Anderson Cancer Center’s Institutional Animal Care and Use Committee guidelines. Six to 8- week old female, C57BL/6 and BALB/c, mice were purchased from Jackson Laboratories (Bar Harbor, ME).

### Bilirubin Nanoparticle

BRNPs were prepared as described previously (12) and lyophilized for long term storage. Briefly, polyethylene glycol-modified bilirubin (PEG-BR) was dissolved in chloroform and dried under nitrogen gas, where it was further dried to generate a film layer. To formulate the nanoparticle, PEG-BR based film was hydrated with phosphate buffered saline (PBS) where it was sonicated for 10 minutes to produce BRNPs. For longer term use, BRNPs were lyophilized and stored at -30°C. Lyophilized BRNPs were resuspended in PBS at a concentration of 5 mg/ml prior to use and stored at 4°C and used within 7 days.

### Indocyanine Green-Encapsulated BRNPs

Indocyanine green (ICG), a near-infrared fluorophore, was loaded in the BRNPs for *in vivo* near-infrared imaging of organs from injected mice using Xenogen IVIS Lumina *in vivo* imaging system. To yield ICG-BRNPs, the film layer of PEG-BR was hydrated using 1 mg/mL aqueous solution of ICG followed by sonication for 15 minutes in the dark (14).

### Murine Bone Marrow Transplantation

Ten to 15- week old female BALB/c mice were irradiated at a myeloablative dose of 8 Gy using ^137^Cs irradiator on day 0. The next day, mice intravenously received a total of 5 x 10^6^ bone marrow cells with or without 5 x 10^6^ splenocytes from age-matched donor female C57BL/6 mice, and with 5 daily intravenous doses of 10 mg/kg BRNP or equivalent volume of vehicle from day 0 to day 4. Subsequently mice were assessed three times weekly for survival and clinical GVHD.

### Evaluation of Clinical GVHD

Clinical GVHD was scored as previously established by Cooke et al., 1996 (17). Briefly, recipient mice were scored from 0-2 for Weight (0: <10% loss, 1: ≥10% <25%, 2: ≥25%), Fur (0: normal, 1: mild to moderate, 2: severe ruffling), Posture (0: normal, 1: kyphosis at rest, 2: kyphosis impairing movement), Activity (0: normal, 1: stationary 50% of the time, 2: stationary unless stimulated), and Skin (0: normal, 1: scaling paws or tails, 2: lesions). Moribund mice were sacrificed according to IACUC guidelines.

### Evaluation of Pathologic GVHD

Recipient mice were sacrificed on day 8 after the transplantation. Organs (spleen, liver, lung, skin, small intestine) and serum was procured, and part of organs were fixed with 10% buffered formalin. Fixed organs were embedded in paraffin and sectioned in 6µm thickness, followed by tissue staining with hematoxylin and eosin. GVHD associated damages were evaluated by pathologist in a blind manner. Pathologic GVHD score was defined as follows: Lung: the presence of perivascular and peribronchial inflammation (ratio) and degree of interstitial pneumonia (1:<25%, 2: <50%, 3: <75%, 4:≥75); Liver: the presence of portal and central vein inflammation (ratio) and number of necrotic foci per view in 10x object, small intestine; number of single cell necrosis per view in 40x object; skin: presence of lymphocyte infiltrates in hair follicles (ratio).

### Flow Cytometry Analysis

Single cell suspension was prepared from liver, spleen, and lung, and stained with antibodies specific for various surface antigens such as CD3ε, CD4, CD8α, CTLA-4, PD-1, ICOS, B220, iNK TCR, and Fixable Viability Stain 620 in PBS for 30 min at 4°C. The cells were then washed with 1X PBS, fixed in 2% paraformaldehyde, and stored at 4°C until acquisition. For intracellular staining of transcription factor – FoxP3 and Helios, cells were fixed and permeabilized using BD Cytofix/Cytoperm buffer (Beckton Dickinson, NJ) for 20 min after surface antigen staining, followed by incubation with antibodies specific for transcription factors in BD Perm/Wash buffer for 45 min. After wash, the samples were acquired using LSRFortessa™ X-20 Cell Analyzer (Beckton Dickinson, NJ), and FlowJo version 10.3 (Tree Star, OR) was used for analysis.

### Serum Cytokine Analysis

First, blood from each individual mouse was collected by retro-orbital vein puncture. The blood from each mouse was placed in tubes to be centrifuged at 600xg for 5 minutes. Serum was collected and stored at -80°C until further analysis. Sera were diluted 1:4 in assay diluent from BD™ Cytometric Bead Array (CBA, Becton-Dickinson, NJ) to determine the serum cytokine concentrations of Th1/Th2/Th17 type cytokines (IL-2, IL-4, IL-6, IFN-γ, TNF-α, IL-17A, and IL-10). The sera were incubated with mixed capture beads and subsequently washed according to the manufacturer’s instructions. Samples were acquired using LSR Fortessa Cell Analyzer (Beckton, Dickinson and Company, Franklin Lakes, NJ), and FlowJo version 10.3 (Tree Star, Ashland, OR) was used for analysis.

### Analysis of Biodistribution of BRNP

On day 0, 10- to 15- week old BALB/c mice received a myeloablative dose of 8 Gy TBI. The following day, mice were intravenously infused with 5 x 10^6^ bone marrow cells with or without 5 x 10^6^ splenocytes isolated from C57BL/6 mice. Additional BALB/c mice received 8 Gy TBI on day 4. On Day 5, mice were intravenously infused with BRNP-ICGs at a single dose of 35 mg/kg per mice and sacrificed 5 hours after infusion. The major organs (spleen, kidney, heart, lungs, liver, small intestine, and large intestine) were harvested to measure fluorescence intensities using a Xenogen IVIS Lumina *in vivo* imaging system (PerkinElmer, MA) with an ICG filter channel and an exposure time of 5 seconds.

### Statistical Analysis

All statistical analyses were performed using GraphPad Prism Software version 8.00 (La Jolla, California) for Windows. Data sets were analyzed using either student *t*-test or Mann-Whitney U tests and p values for comparisons between groups were determined. For survival analysis, Log-rank (Mantel-Cox) survival test was used. P value less than 0.05 was considered to be statistically significant.

## Results

### BRNPs Selectively Accumulate at GVHD Target Organs

**Figure 1:**
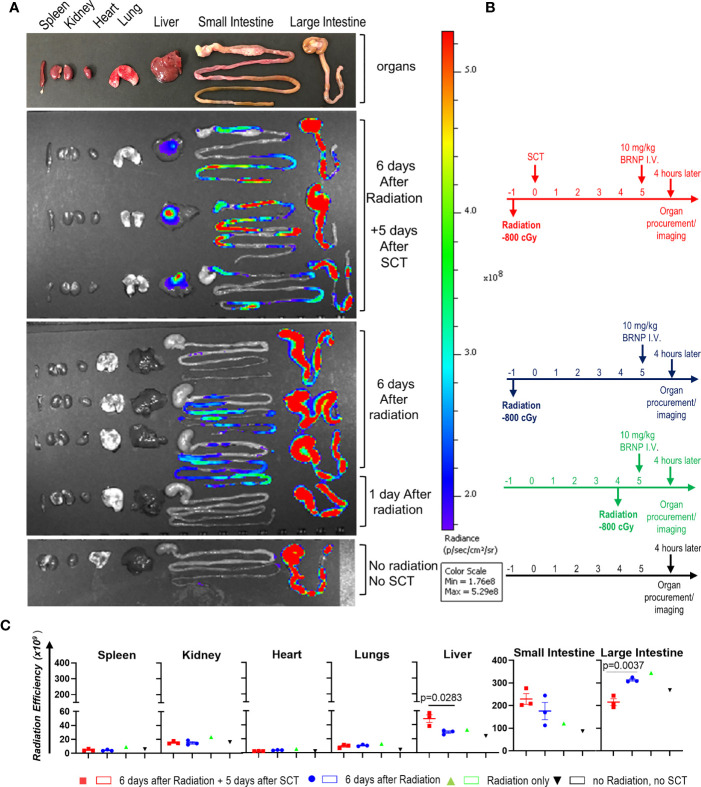
BRNPs accumulate GVHD target organs. **(A)** Representative intensity imaging of ICG-BRNP infiltration on organs from mice without total body irradiation (TBI) and mismatched donor bone marrow transplantation, 1 day or 6 days after 8Gy TBI only, and 6 days after 8Gy TBI in addition to 5 days after mismatched donor bone marrow transplantation, and no radiation, no transplantation (SCT) or BRNP-ICG infusion (control). **(B)** Representative time graphs of organ procurement and radiance imaging per treatment group. **(C)** Radiance intensity of each organs isolated from mice assigned to different treatment group. There was a physiologic accumulation of ICG-BRNPs in colon, and increased ICG-BRNPs accumulation especially in liver and small bowel after radiation followed by BMT. Each dot represents value from single organ. The error bars represent standard deviation. Mann-Whitney U test was used to compare differences between two groups. P-value less than 0.05 was considered “significant”, and only significant values were presented.

Previous studies have demonstrated that BRNPs selectively accumulate at the area of inflammation elicited by various tissue injury from ischemia, allergen, dextran sodium sulfate and as well as autoimmune processes (10–15). As acute GVHD cascade involves systemic and local inflammation caused by conditioning regimen and infiltrating donor immune cells, we sought to investigate whether BRNPs preferentially accumulate in the area of initial tissue injury – especially in the liver and gut from conditioning regimen of radiation commonly used in allogeneic stem cell transplantation. In bone marrow transplantation (BMT), alloreactive memory T cells or primed naïve T cells may start proliferating from 3-5 days after transplantation, further magnifying the inflammation from initial tissue injury, leading to detectable pathologic findings of GVHD as early as day 7. Therefore, we sought to demonstrate the biodistribution of BRNPs at day 6 after conditioning regimen with total body irradiation (TBI) at a myeloablative dose followed by MHC mismatched donor cell transplantation (**Figure 1**).

First, there was a physiologic accumulation of ICG-BRNPs in colon through the course. ICG-BRNPs were detectable in the small intestine of recipient mice, 6 days after TBI, and significantly increased in mice after major MHC mismatched BMT. ICG-BRNP accumulation was observed in additional GVHD-target organ, i.e. liver of the mice only after BMT, and was not detectable in the non-GHVD target organs, that is spleen, kidney, and heart of both the groups (**Figure 1B**). These results support that BRNP preferentially accumulates at GVHD-target organs after the BMT.

### BRNPs Improve Clinical GVHD and GVHD Related Mortality

Previous *in vivo* pharmacokinetics study demonstrated that half-life of BRNPs is 9.3 hours with a peak level of bilirubin in serum being 100 mg/dl immediately after BRNPs at 50 mg/kg was intravenously infused (11). As serum bilirubin can increase in setting of liver injury from conditioning regimen and GVHD post transplantation (1–3), we chose BRNPs given to recipient mice intravenously daily at a dose of 10 mg/kg from day 0 to day 4 to allow near normalization of serum bilirubin level by day 7 or 8. Moreover, single or multiple infusions of BRNP at a dose of 10 mg/kg has been shown to effectively prevent hepatic ischemic perfusion injury (10), and pancreatic islet xenotransplantation (16), supporting our dose and dosing strategy.

**Figure 2:**
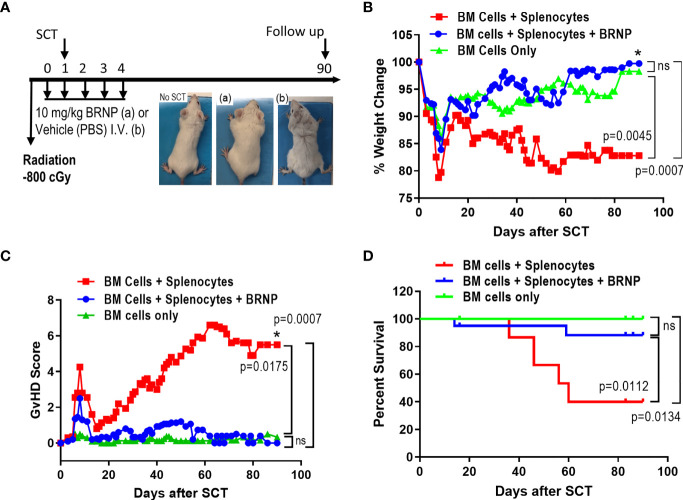
BRNP treatment improves clinical GVHD and GVHD-related mortality. **(A)** Overall treatment scheme. Briefly, BALB/c recipient mice received Total Body Irradiation (TBI) at a dose of 8Gy on day 0, followed by transplantation with donor bone marrow (▲, n=9) or bone marrow plus splenocytes on day 1 (■, n=20). Part of recipients of bone marrow plus splenocytes received BRNPs at a dose of 10 mg/kg daily from days 0 to 4 (●, n=20). Mice were monitored for weights **(B)**, activity, fur, skin, posture – signs for clinical GVHD **(C)** three times a week, and survival **(D)** for 90 days. BRNP treatment significantly reduced clinical course of GVHD and subsequently GVHD-related mortality after mismatched donor bone marrow transplantation. Two independent experiments were performed, and results were combined for the final analysis. Mann-Whitney U test was used to compare values on day 90 between two groups **(B, C)**, and Log-Rank test was used to compare survival between two groups **(D)**, P-value less than 0.05 was considered “significant”, and “ns” indicated “non-significance”. The black asterisk (*) refers to significant p-values.

After myeloablative irradiation, BALB/c mice (MHC d-haplotype: H-2^d^, IA/E^d^) received 5x10^6^ bone marrow cells isolated from C56BL/6 on day 1 to reconstitute recipient immune system, or 5x10^6^ splenocytes in addition to 5x10^6^ bone marrow cells to induce graft versus host disease. Portion of these mice were infused with BRNP at a dose of 10 mg/kg or vehicle control daily from day 0 to day 4 (**Figure 2A**). Mice were monitored for clinical GVHD (weight, fur, activity, posture, and skin) and survival for 90 days. We observe transient weight loss and corresponding increase in GVHD score around day 7, which was related to radiation treatment (**Figure 2B**). As expected, recipient mice with BMT only did not develop clinical GVHD and survived through day 90 from BMT (**Figure 2C**), but mice infused with a mixture of splenocytes and bone marrow cells started to develop progressive clinical signs of GVHD around day 14-such as ruffled fur, hunched back, decreased activity, and weight loss compared to BMT control (p=0.0007) with significant GVHD-related mortality (HR 5.898, p=0.0134). Treatment with BRNP prevented recipient mice from developing clinical GVHD and resulted in improved overall survival compared to vehicle treatment group (HR 0.2055, p=0.0112), demonstrating that targeting early inflammation cascade with BRNP is feasible and has therapeutic benefits in BMT (**Figure 2D**).

### BRNPs Reduce Tissue Damages From Graft Versus Host Disease 

**Figure 3:**
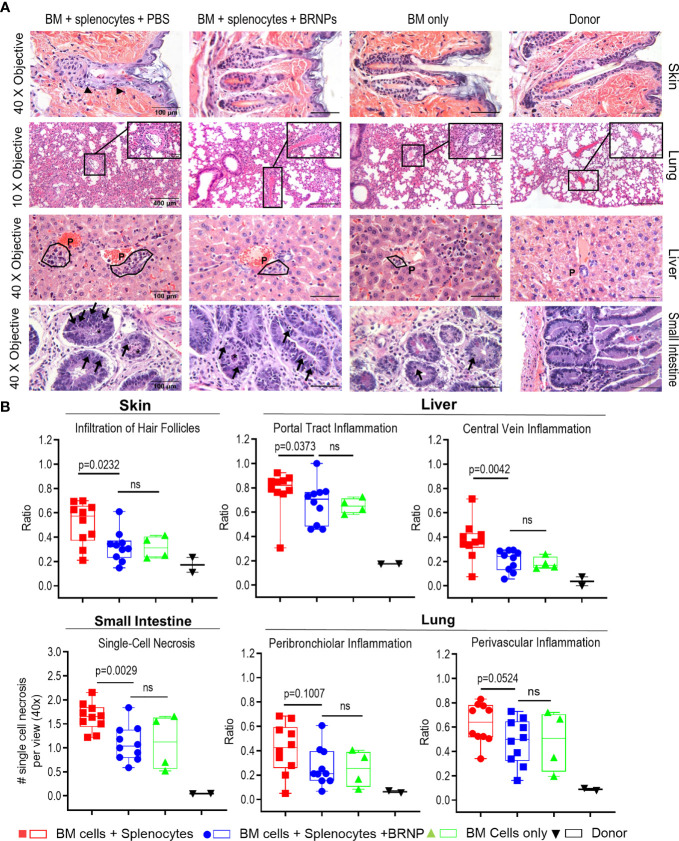
BRNPs reduce tissue damages from graft versus host disease. **(A)** Representative images of hematoxylin and eosin (H&E) staining of GVHD target organs (liver, skin, small intestine of gastrointestinal track), and lung. Black arrows in skin show examples of inflammatory cells infiltrated around hair follicles. The symbol “P” in the liver noted example of portal track. Black arrows in small intestine show single cell necroses in the glandular epithelium and the black asterisk (*) show necrotic cells in crypt lumen. **(B)** Summary of histopathologic findings from GVHD organs from donor (▼, n=2), and transplant recipient mice with bone marrow (▲, n=4), bone marrow and splenocytes (■, n=10), bone marrow and splenocytes plus BRNPs (●, n=10). BRNP treatment significantly reduced histopathologic signs of tissue damage in most GVHD organs. Two independent experiments were performed, and results were combined for the final statistical analysis. Each dot represents a value from a single mouse. The error bars represent standard deviation. Statistical analysis was performed using Mann-Whitney U Test. P-value less than 0.05 was considered “significant”, and “ns” indicated “non-significance”.

The initial tissue injury may start from conditioning chemotherapy or radiation in transplantation, but subsequent damage to organ mediated by alloantigen specific donor T cells is detrimental. In murine BMT model, GVHD related tissue damage can be detectable as early as day 7 post transplantation. To investigate whether BRNPs rescue recipient mice from GVHD-related tissue damage, we performed histopathological analyses on GvHD target organs such as the liver, lung, gastrointestinal tract (GI), and skin on recipient mice at 8 days after major MHC-mismatched BMT (**Figure 3A**).

We observed a significantly lower inflammatory immune cell infiltration in hair follicles of skin (p=0.0232), less inflammation in the portal tracks and central veins of liver (p=0.0373, p=0.0042, respectively), less single cell necrosis in small intestine (p=0.0029), and a trend of decrease in peribronchiolar and perivascular inflammation in lung (p=0.1007, p=0.0524, respectively) from BRNP treated mice when compared to the mice treated with vehicle (**Figure 3B**). Of note, histopathologic finding of tissue injury from recipient mice treated with BRNP did not have significant differences from recipient mice with BMT only, demonstrating that BRNP treatment peri transplantation could prevent histopathologic GVHD of target organs.

### BRNPs Reduce Serum Level of Proinflammatory Cytokine, IFN-γ

**Figure 4:**
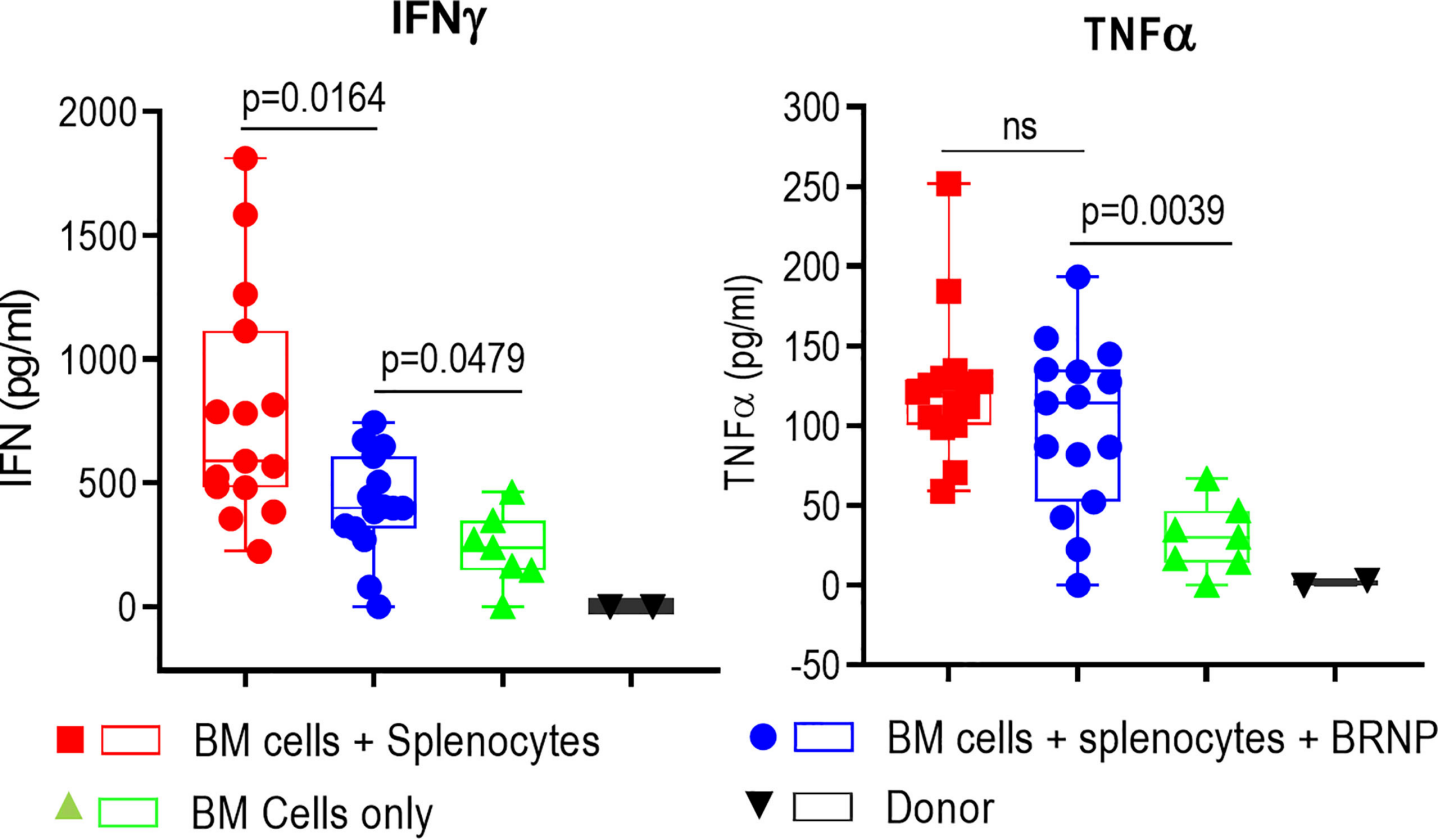
BRNPs reduce systemic inflammatory responses. Two representative inflammatory cytokines were assessed in the sera from donor (▼, n=2), and transplant recipient mice with bone marrow (▲, n=7), bone marrow and splenocytes (■, n=15), bone marrow and splenocytes plus BRNPs (●, n=15) 8 days after transplantation. BRNP treatment significantly reduced *in vivo* production of IFN-γ but marginally of TNF-α. Three independent experiments were performed, and results were combined for final statistical analysis using Mann-Whitney U Test. Each dot represents a value from a single mice. The error bars represent standard deviation. P-value less than 0.05 was considered “significant”, and “ns” indicated “non-significance”.

Inflammatory cytokines such as IL1-β, TNF-α, IL-6, IFN-γ, etc., are first produced by innate immune cells in response to tissue damage caused by conditioning regimen, this inflammatory responses is further augmented once alloantigen specific T cells are activated and expanded in the area of damaged tissue (8). Previous studies indicate higher serum levels of these pro-inflammatory cytokines are associated with higher organ damage causing more severe onset of acute GVHD (18). We also observed significantly higher levels of inflammatory cytokines such as IFN-γ and TNF-α (**Figure 4**) in sera from mice with GVHD than those from mice without GVHD as early as 8 days post transplantation, confirming systemic inflammatory responses occurred during GVHD. Another proinflammatory cytokine, IL-6, was detectable but there was no difference between mice with and without GVHD, likely due to the time of evaluation (data not shown). The sera level of IFN-γ was significantly reduced in mice treated with BRNPs compared to mice without BRNPs (p=0.0164) but still elevated compared to mice with BMT only (p=0.0479). In contrast, there was no difference in the serum of TNF-α in mice treated with BRNPs compared to untreated mice with GVHD, and still significantly elevated compared to mice with BMT only (p=0.0039). Our results indicate that BRNP treatment peri-transplantation reduced the sera level of IFN-γ, a potent proinflammatory cytokines produced by lymphoid cells (19).

### BRNPs Reduce the Infiltration of Neutrophils and CD8α^+^ iNK T Cells, and Activation of Donor T Cells 

**Figure 5:**
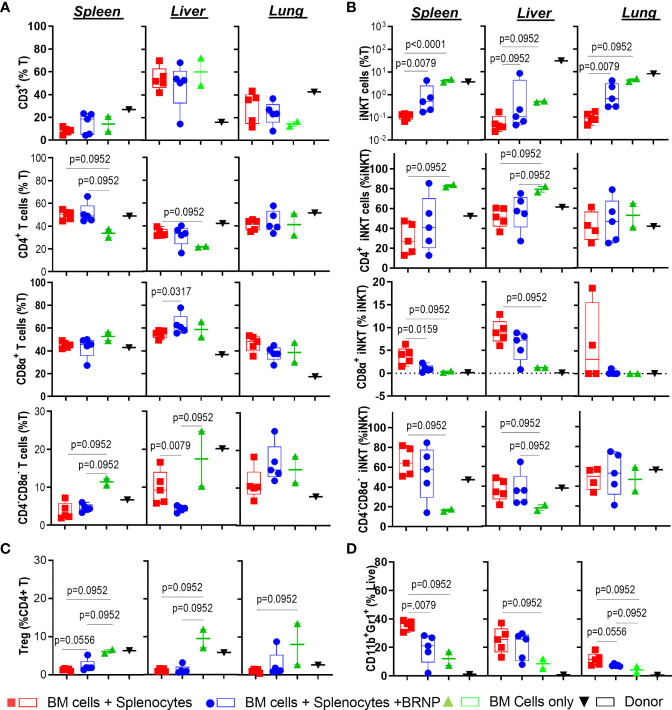
BRNPs reduce the infiltration of neutrophils and CD8α^+^ iNK T cells. Multi-parameter flowcytometric analysis of immune subsets –T cells **(A)**, invariant Natural Killer T (iNK T) cells **(B)**, conventional regulatory T cells (Treg) **(C)**, neutrophils **(D)** from spleen, liver, and lung of donor (▼, n=1), and transplant recipient mice with bone marrow (▲, n=2), bone marrow and splenocytes (■, n=5), bone marrow and splenocytes plus BRNPs (●, n=5) 8 days after transplantation. BRNP treatment helped the reconstitution of iNK T cells systemically and locally, especially immunoregulatory CD4^+^ iNK T cells with reciprocal reduction of CD8α^+^ iNK T cells, inflammatory subtype. In contrast, BRNP reduced the expansion or infiltration of neutrophils in particular in liver. Two independent experiments were performed, and one representative data were presented. Each dot represents a value from a single mouse. The error bars represent standard deviation. Mann-Whitney U Tests were used for statistical analysis. P-value less than 0.05 was considered “significant”, and only significant P values were noted.

GVHD is a disease of dysregulated donor T cells where naïve T cells are inadvertently primed to recognize alloantigens released during initial tissue injury, and subsequently mediate further tissue damage in transplant recipients. As we observed that BRNP treatment significantly improved clinical GVHD and GVHD-related mortality by the reduction of local tissue damage and systemic inflammatory cytokines, we investigated whether BRNP treatment prevents the early recruitment of donor derived T cells along with various inflammatory or regulatory immune cells such as neutrophils (CD11b^+^GR1^+^), invariant Natural Killer T cells (iNK T, αGalCer/CD1d tetramer^+^) or regulatory T cells (Treg, CD4^+^FoxP3^+^Helios^+^) 8 days after major MHC mismatched BMT among mice that received BMT only for normal hematopoiesis, bone marrow plus splenocytes for inducing GVHD, and bone marrow plus splenocytes with BRNP treatment (**Supplementary Figure 1**). First, we did not see significant differences in frequencies of T cells or consistent differences of major T cells type – CD4^+^, CD8α^+^, CD4^-^CD8α^-^ T cells systemically in spleen or locally in lung or liver, between mice developing GVHD with or without BRNP treatment (**Figure 5A**). However, there was a significantly delayed reconstitution of another innate regulatory immune cells, iNK T cells, in mice developing GVHD, and BRNP treatment increased the frequency of iNK T cells, in spleen and lung and reduced in liver (**Supplementary Figure 2**). Interestingly, CD8α^+^ iNK T cells, an effector/inflammatory subset of iNK T cells, were significantly increased in spleen, and locally in liver in mice developing GVHD (p = 0.0952, p = 0.0952, respectively), with a reciprocal decrease in CD4^+^ iNK T cells, regulatory subset of iNK T cells (p = 0.0952, p = 0.0952, respectively), compared to mice with BMT only. BRNP treatment peri-transplantation significantly blunted the expansion of CD8α^+^ iNK T cells in spleen and liver and let to restoring CD4^+^ iNK T subsets (**Figure 5B**). Of note, mice developing GVHD had significantly lower frequencies of conventional Tregs systemically in spleen and locally in liver and lung compared to mice with normal hematopoiesis, and there was a trend towards an increase in splenic Treg frequencies with BRNP treatment (**Figure 5C**). Lastly, there was an expansion of neutrophils in the spleen, locally in liver and lung of mice with GVHD compared to those of mice with normal hematopoiesis. Again, BRNP treatment reduced infiltration of neutrophils compared to mice with no treatment (**Figure 5D**).

**Figure 6:**
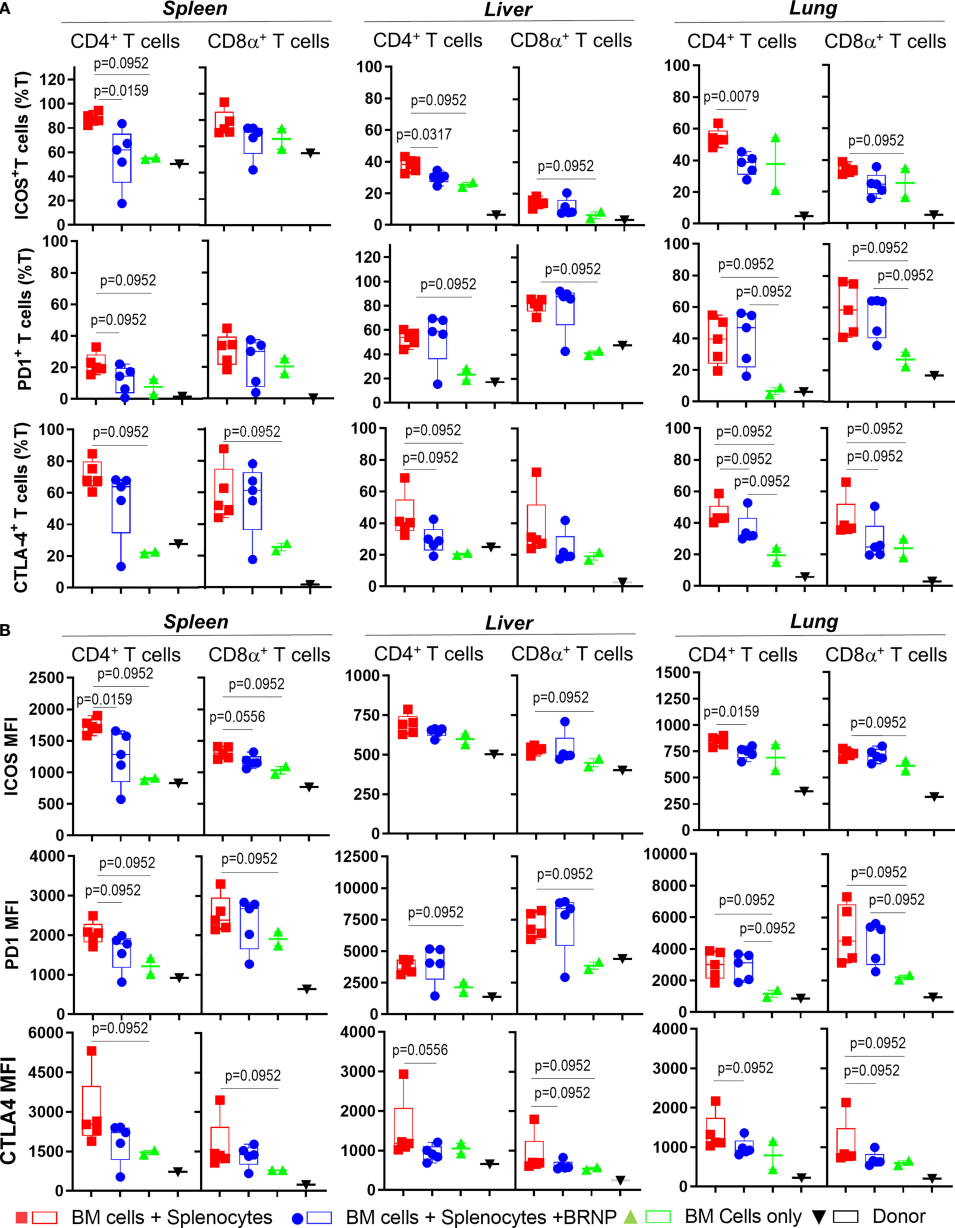
BRNPs reduce systemic and local activation of donor derived T cells. Multi-parameter flowcytometric analysis of Inducible T cell costimulatory (ICOS), PD-1, and CTLA-4 of T cells from spleen, liver, and lung of donor (▼, n=1), and transplant recipient mice with bone marrow (▲, n=2), bone marrow and splenocytes (■, n=5), bone marrow and splenocytes plus BRNPs (●, n=5) 8 days after transplantation. Percent ICOS^+^, PD-1^+^, and CTLA-4^+^ CD4^+^ or CD8α^+^ T cells were presented in **(A)**, and mean fluorescent intensity of ICOS, PD-1, and CLTA-4 expression on CD4^+^ or CD8α^+^ T cells were indicated in **(B)**. ICOS^+^ or CLTA-4^+^ T cells and less markedly PD-1^+^ T cells were significantly increased systemically and locally in mice transplanted with bone marrow and splenocytes, and these increases were partially blunted with BRNP treatment in mostly ICOS^+^ and CTLA-4^+^ CD4^+^ T cells. BRNP treatment did not significantly affect the expression of PD-1 on T cells locally in liver and lung. Two independent experiments were performed, and one representative data were presented. Each dot represents a value from a single mouse. The error bars represent standard deviation. Mann-Whitney U Tests were used for statistical analysis. P-value less than 0.05 was considered “significant”, and only significant P values were noted.

Although we did not see consistent alteration in major T cell subsets associated with GVHD, we observed significant upregulation in Inducible T cell Costimulator (ICOS) in both CD4^+^ and CD8α^+^ T cell subsets in mice developing GVHD compared to those with normal hematopoiesis, and the expression of ICOS was reduced after BRNP treatment (**Figure 6**). Likewise, immune checkpoint inhibitors, - PD-1 but not CLTA-4 were highly upregulated in T cells from mice developing GVHD compared to those with normal hematopoiesis but BRNP treatment reduced PD-1 expression only in splenic T cells (**Supplementary Figure 2**).

Altogether, we demonstrated that inflammatory cells such as neutrophils or CD8α^+^ iNK T cells were significantly infiltrated, locally in mice with GVHD, and BRNP treatment blunted expansion of neutrophils and CD8α^+^ iNK T cells. Although there was no significant alteration of major conventional T cell subsets, there was significant upregulation of ICOS and PD-1 in T cells during GVHD, and again the expression of these proteins was reduced with BRNP treatment, more in splenic T cells than liver or lung residential T cells. Our findings suggest that BRNP treatment ameliorates GVHD-related tissue damage *via* dampening early activation of conventional T cells and infiltration of inflammatory innate immune cells.

## Discussion

Conventional approach to prevent acute GVHD after allogeneic stem cell transplantation is the use of immunosuppressants such as calcineurin inhibitors (tacrolimus or cyclosporine in combination of methotrexate or mycophenolate with or without rabbit anti-thymocyte globulin antibody (ATG), and more recently post-transplantation cyclophosphamide with or without calcineurin inhibitors and mycophenolate (20). These primary prophylactic treatments universally aim at regulating the activation and expansion of alloantigen reactive donor T cells, hope to reduce donor T cell mediated damages and recruitment of additional immune subsets that may substantiate further tissue injury (5, 8). Despite recent advances in the understanding of pathophysiology of acute GVHD, most therapeutics that have been evaluated in preclinical and clinical settings target the dysregulated donor T cell responses and subsequent adaptive inflammatory milieu (7, 8).

Reactive oxygen species (ROS) are thought to play a key role in the progression of inflammation and tissue injury (21), and there are a few lines of evidence supporting the role in the pathogenesis of acute GVHD. While the pharmacologic blockade of NADPH oxidase complex to reduce the production of ROS after transplantation ameliorated clinical GVHD and survival (22), the deficiency of Indoleamine 2,3-dioxygenase 1 (IDO1), scavengers for ROS, in donor cells exacerbated GVHD. More directly, cyclopentylamino carboxymethylthiazolylindole (NecroX)-7 has attenuated acute GVHD by inhibiting the formation of mitochondria specific ROS/reactive nitrogen species, in turn, preventing the release of High-mobility group box 1 (HMGB1) (23). These studies demonstrate that it may be feasible to intervene the ROS formation from donor cells as well as initial tissue injury, thus preventing acute GVHD.

Bilirubin, a final product of heme catabolism, is an effective antioxidant associated with protection against cardiovascular diseases, non-alcoholic fatty liver disease, colorectal cancer, ischemia–reperfusion injury after liver transplantation (24), as well as experimental autoimmune encephalomyelitis (25, 26). However, a high level of unconjugated bilirubin can accumulate in brain and cause irreversible neurological damage, thus limiting the use as immunomodulant for several disease. Therefore, various strategies to solubilize bilirubin have been attempted to maximize its immunomodulatory effects while minimizing toxicity due to water-insolubility (27). PEGylation of bilirubin is one such strategy to solubilize bilirubin where polyethylene glycol is conjugated with bilirubin, creating an amphiphilic molecule that self-assembles to form stable, non-toxic nanoparticles. Since polyethylene glycol is an FDA approved active ingredient, PEGylation is often utilized to improve the pharmacokinetic activity of various FDA approved drugs such as PEG-interferon alfa-2a for hepatitis C treatment (28). Likewise, PEGylation of bilirubin also demonstrated to not affect the characteristic antioxidative and light sensitive properties of bilirubin, in both *in vitro* experiments and various mouse models of inflammatory diseases (10–16).

In current work, we first showed that BRNPs selectively accumulate in GVHD target organs such as small intestine and liver after myeloablative radiation followed by donor stem cell transplantation. Further, five daily treatments with BRNPs at 10 mg/kg peri- and post- transplantation can effectively alleviate clinical and histopathologic GVHD and significantly improve GVHD related mortality. Accordingly, there was a significant decrease in IFN-γ, one of proinflammatory cytokine that is produced by lymphoid cells such as Th1 CD4^+^ T Cells, cytotoxic CD8α^+^ T cells, NK cells, and innate lymphoid cells (ILC) (19), and most importantly alloreactive T cells during GVHD (29), but not in TNFα, a major cytokine produced mostly by monocyte lineage immune cells such as macrophages (8). At cellular level, bilirubin treatment reduced the early infiltration of inflammatory immune cells such as GR1^+^ CD11b^+^ neutrophils and CD8α^+^ iNK T cells and facilitated the recovery of regulatory CD4^+^ iNK T cells. Although systemic T cell proliferation or local infiltration to GVHD organs was not significantly altered after BRNP treatment, the expression of ICOS and CTLA-4 on donor T cells as well as frequencies of- ICOS^+^ or CLTA-4^+^ donor T cells were reduced with BRNP treatment, suggesting that BRNP treatment may have blunted the activation of alloantigen reactive donor T cells post transplantation. Our findings indicated that BRNPs can not only control early inflammation but also influence subsequent adaptive donor T cell immunity to mitigate acute GVHD.

Interestingly, previous investigation of BRNPs on immune subsets are similar to our observation in that BRNP treatment consistently reduced the infiltration of GR1^+^ CD11b^+^ neutrophils to liver during ischemia-reperfusion injury, and lung in a murine bleomycin-induced pulmonary fibrosis model (10, 14). Differently from these reports, it is noteworthy that BRNP treatment did not completely reverse histopathologic findings, inflammatory cytokine production, infiltration of inflammatory immune cells, as well as activated donor T cells in our murine GVHD model. In transplantation, the activation of donor T cells in tumor antigen specific manner is critical to mediate the graft versus tumor (GVT) effects of donor T cells, thus a certain degree of residual inflammation after BRNP treatment may be essential to preserve GVT effects of donor T cells. Further investigation is needed to demonstrate the impact of BRNPs on GVHD vs GVT effects of donor T cells.

One unique property of BRNPs over other ROS scavengers is their ability to capture small molecules and form drug-BRNP complex, accumulate in the area of inflammation, and release drugs in response to ROI that are abundantly present in tumor microenvironment (30, 31). Thus, BRNPs can potentially increase bioavailability and therapeutic efficacy of conjugated small molecules. Systemic steroid or additional immunosuppressants (ISs) such as ruxolitinib, ibrutinib, itacitinib, pentostatin are currently being used to treat acute or chronic GVHD, and their global immune-suppression can not only blunt beneficial graft versus tumor effects of donor T cells but also cause various infections leading to increase non-treatment related mortality after transplantation (1–3). Therefore, IS-BRNPs may have potential therapeutic benefits over BRNPs or ISs alone as they will selectively accumulate to the GVHD organs, where BRNPs can scavenge ROS and released IS will suppress dysregulated donor immune cells present in GVHD organs. Further investigation warrants development of BRNP-based immune-modulator to prevent and treat GVHD post transplantation.

## Data Availability Statement

The raw data supporting the conclusions of this article will be made available by the authors, without undue reservation.

## Ethics Statement

The animal study was reviewed and approved by institutional animal care and use committee at the University of Texas M.D. Anderson Cancer Center.

## Author Contributions

SP, AF, and JI designed the experiments. SP, AF, DB, JK, DY, AT-O, S-EL, and MZ performed experiments. SP, AF, and JI interpreted results. SP, AF, SJ and JI wrote the manuscript. All authors contributed to the article and approved the submitted version.

## Funding

The research was supported by MDACC Institutional Research Grant and Start-Up Fund (JI), the South Campus Flow Cytometry and Cell Sorting Core (NCI # P30CA016672) and by the Basic Science Research Program of the National Research Foundation of Korea (NRF2018R1A3B1052661) funded by the Ministry of Science and ICT (SJ).

## Conflict of Interest

The authors declare that the research was conducted in the absence of any commercial or financial relationships that could be construed as a potential conflict of interest.

## Publisher’s Note

All claims expressed in this article are solely those of the authors and do not necessarily represent those of their affiliated organizations, or those of the publisher, the editors and the reviewers. Any product that may be evaluated in this article, or claim that may be made by its manufacturer, is not guaranteed or endorsed by the publisher.

## Supplementary Material

Supplementary Figure 1 Representative Flowcytometric analysis of various immune subsets of splenocytes isolated from mice who received BM and splenocytes, BM and splenocytes with BRNP treatment, BM only and donor cells only. **(A)** A gating strategy for T cells, iNK T cells, Treg, and expression of CD4 and CD8a on T cells. First, lymphocytes were selected according to FSC and SSC, and subsequently dead cells were excluded. The iNK T cells, and T cells were selected by expression of iNK-TCR (aGalCer/CD1d+) and CD3, or CD3 alone respectively. The expression of CD4 or CD8a were assessed from iNK T cells or T cells. Conventional regulatory T cells were defined as FoxP3^+^Helios^+^ cells from CD4^+^ T cells. **(B)** A gating strategy for neutrophils CD11b^+^GR1^+^ neutrophils. Dead cells were excluded from mononuclear cells, and CD11b^+^GR1^+^ neutrophils were assessed from CD3^-^B220^-^ population. **(C)** Representative analysis of checkpoint inhibitors: ICOS, PD1, CTLA4 with a significant upregulation of all check point inhibitors in mice received BM + splenocytes, compared to mice received BM only or donor. A single representative sample was taken from each of the following groups to form the tSNE flowcytometric analysis.

Supplementary Figure 2 Flowcytometric tSNE plots of various immune subsets of splenocytes, liver lymphocytes and lung lymphocytes isolated from mice received BM and splenocytes, BM and splenocytes with BRNP treatment, BM only, and donor cells only. Parameter tSNE flowcytometric analysis of Immune subsets of **(A)** CD4^+ (^blue), CD8α^+^ (orange), CD4^-^CD8α T cells (light green), conventional T regulatory cells (dark green) and **(B)** CD4^+^(blue), CD8α^+^ (orange), CD4^-^CD8α T cells (light green), α−GalCer Tetramer (dark green) from spleen, liver, and lung of donor, and transplant recipient mice with bone marrow, bone marrow and splenocytes, bone marrow and splenocytes plus BRNPs 8 days after transplantation. A single representative sample was taken from each of the following groups to form the tSNE flowcytometric analysis.

Supplementary Figure 3 Flowcytometric tSNE plots of various checkpoint inhibitors of splenocytes, liver lymphocytes and lung lymphocytes isolated from mice who received BM and splenocytes, BM and splenocytes with BRNP treatment, BM only, and donor cells only. Parameter tSNE flowcytometric analysis of Immune subsets of **(A)** ICOS, **(B)** PD-1 and **(C)** CTLA-4 from spleen, liver, and lung of donor, and transplant recipient mice with bone marrow, bone marrow and splenocytes, bone marrow and splenocytes plus BRNPs, 8 days after transplantation. A single representative sample was taken from each of the following groups to form the tSNE flowcytometric analysis.
