# Supplementary figures and images for: Post Transplantation Bilirubin Nanoparticles Ameliorate Murine Graft Versus Host Disease via a Reduction of Systemic and Local Inflammation
Source: Front Immunol. 2022 Jun 1;13:893659. doi: 10.3389/fimmu.2022.893659 (PMC9199387; doi:10.3389/fimmu.2022.893659)

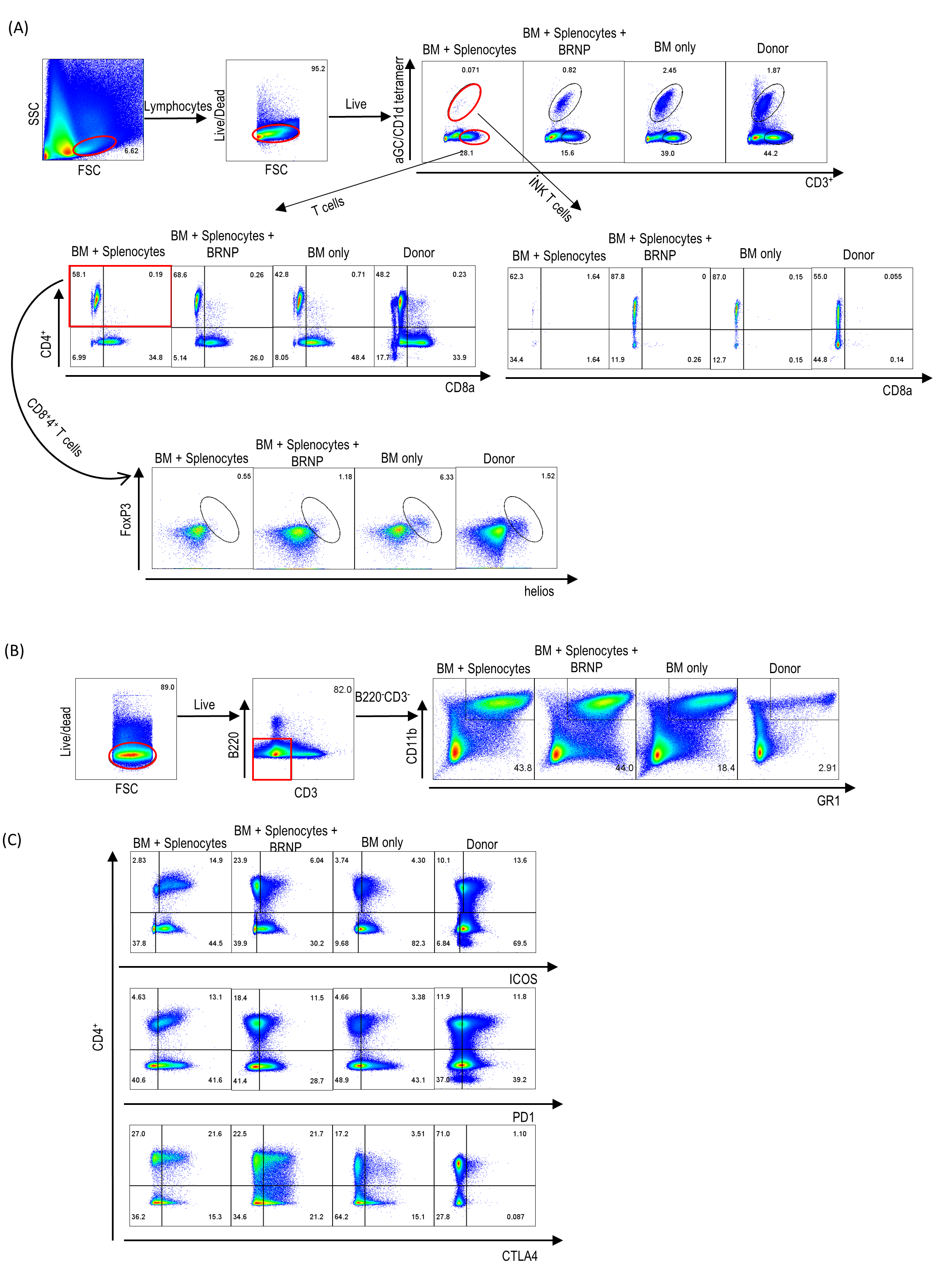

Supplement: Supplementary Figure 1 — Representative Flowcytometric analysis of various immune subsets of splenocytes isolated from mice who received BM and splenocytes, BM and splenocytes with BRNP treatment, BM only and donor cells only. (A) A gating strategy for T cells, iNK T cells, Treg, and expression of CD4 and CD8a on T cells. First, lymphocytes were selected according to FSC and SSC, and subsequently dead cells were excluded. The iNK T cells, and T cells were selected by expression of iNK-TCR (aGalCer/CD1d+) and CD3, or CD3 alone respectively. The expression of CD4 or CD8a were assessed from iNK T cells or T cells. Conventional regulatory T cells were defined as FoxP3+Helios+ cells from CD4+ T cells. (B) A gating strategy for neutrophils CD11b+GR1+ neutrophils. Dead cells were excluded from mononuclear cells, and CD11b+GR1+ neutrophils were assessed from CD3-B220- population. (C) Representative analysis of checkpoint inhibitors: ICOS, PD1, CTLA4 with a significant upregulation of all check point inhibitors in mice received BM + splenocytes, compared to mice received BM only or donor. A single representative sample was taken from each of the following groups to form the tSNE flowcytometric analysis. [file Image_1.tif]

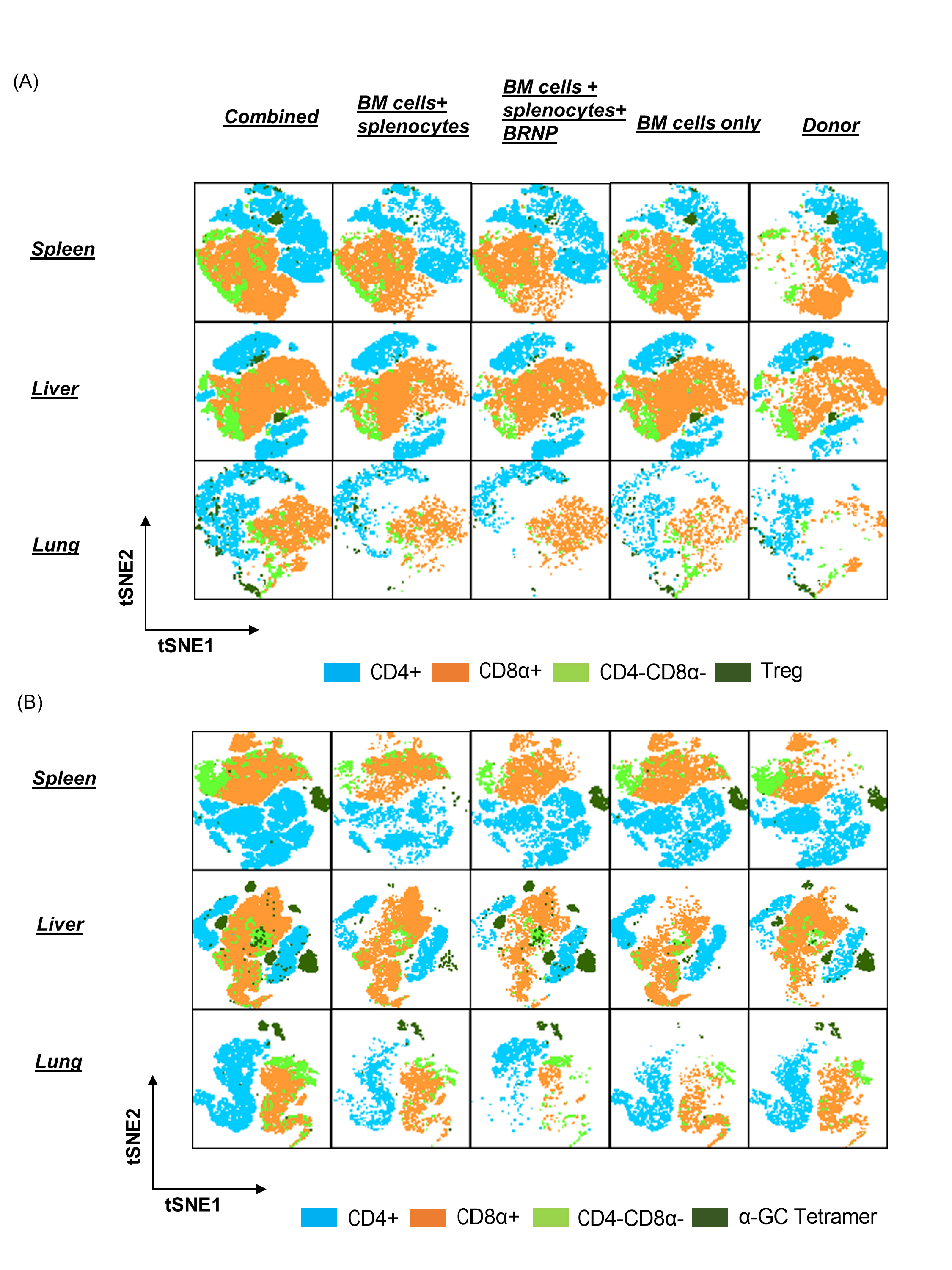

Supplement: Supplementary Figure 2 — Flowcytometric tSNE plots of various immune subsets of splenocytes, liver lymphocytes and lung lymphocytes isolated from mice received BM and splenocytes, BM and splenocytes with BRNP treatment, BM only, and donor cells only. Parameter tSNE flowcytometric analysis of Immune subsets of (A) CD4+ (blue), CD8α+ (orange), CD4-CD8α T cells (light green), conventional T regulatory cells (dark green) and (B) CD4+(blue), CD8α+ (orange), CD4-CD8α T cells (light green), α−GalCer Tetramer (dark green) from spleen, liver, and lung of donor, and transplant recipient mice with bone marrow, bone marrow and splenocytes, bone marrow and splenocytes plus BRNPs 8 days after transplantation. A single representative sample was taken from each of the following groups to form the tSNE flowcytometric analysis. [file Image_2.tif]

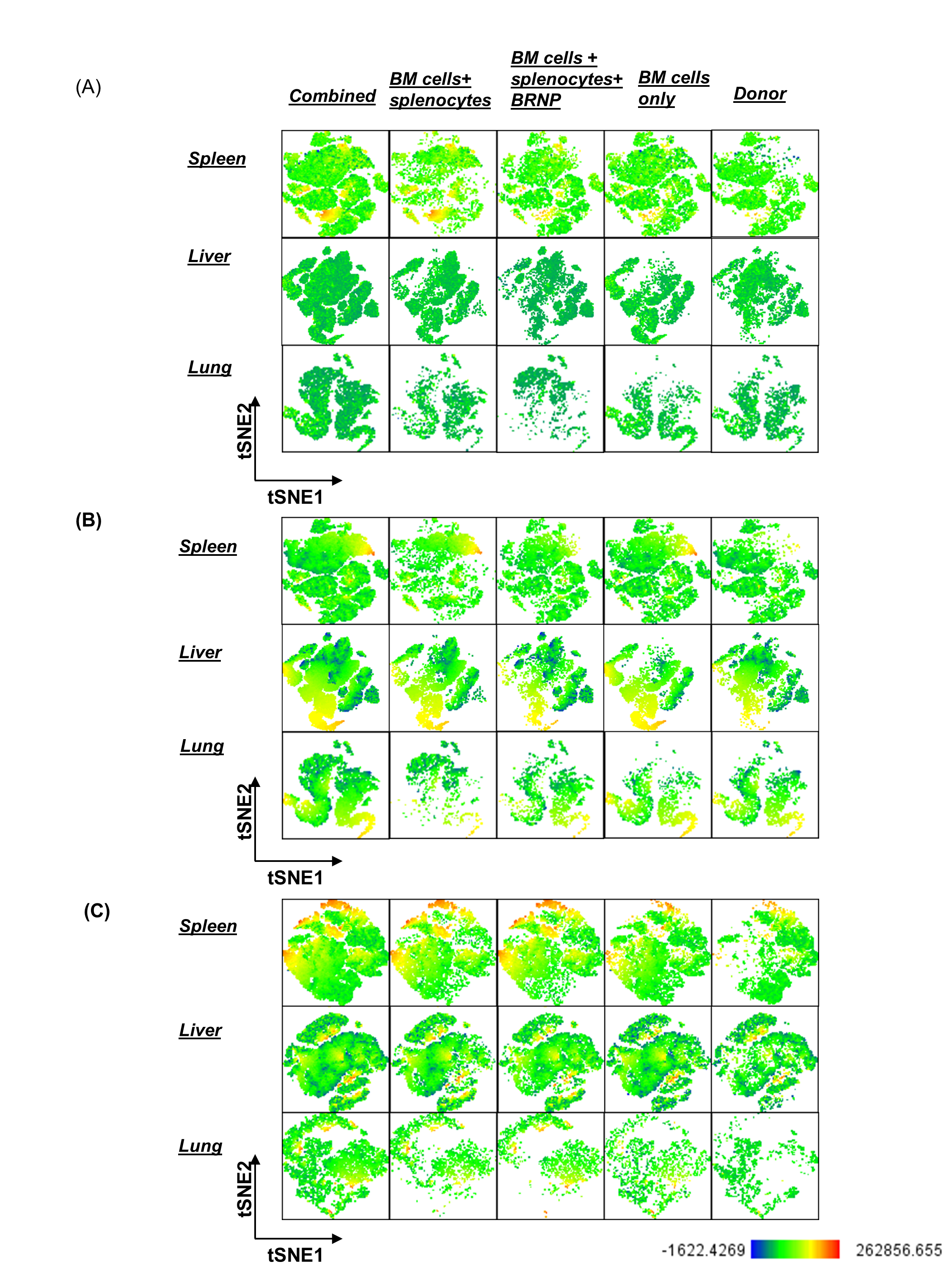

Supplement: Supplementary Figure 3 — Flowcytometric tSNE plots of various checkpoint inhibitors of splenocytes, liver lymphocytes and lung lymphocytes isolated from mice who received BM and splenocytes, BM and splenocytes with BRNP treatment, BM only, and donor cells only. Parameter tSNE flowcytometric analysis of Immune subsets of (A) ICOS, (B) PD-1 and (C) CTLA-4 from spleen, liver, and lung of donor, and transplant recipient mice with bone marrow, bone marrow and splenocytes, bone marrow and splenocytes plus BRNPs, 8 days after transplantation. A single representative sample was taken from each of the following groups to form the tSNE flowcytometric analysis. [file Image_3.tif]
